# Whether weather matters: Evidence of association between in utero meteorological exposures and foetal growth among Indigenous and non-Indigenous mothers in rural Uganda

**DOI:** 10.1371/journal.pone.0179010

**Published:** 2017-06-07

**Authors:** Sarah MacVicar, Lea Berrang-Ford, Sherilee Harper, Yi Huang, Didacus Namanya Bambaiha, Seungmi Yang

**Affiliations:** 1 Department of Geography, McGill University, Montréal, Quebec, Canada; 2 Department of Population Medicine, University of Guelph, Guelph, Ontario, Canada; 3 Department of Atmospheric & Oceanic Sciences, McGill University, Montréal, Quebec, Canada; 4 Ministry of Health, Kampala, Uganda; 5 Department of Epidemiology, Biostatistics, and Occupational Health, McGill University, Montréal, Quebec, Canada; University of Missouri Columbia, UNITED STATES

## Abstract

Pregnancy and birth outcomes have been found to be sensitive to meteorological variation, yet few studies explore this relationship in sub-Saharan Africa where infant mortality rates are the highest in the world. We address this research gap by examining the association between meteorological factors and birth weight in a rural population in southwestern Uganda. Our study included hospital birth records (n = 3197) from 2012 to 2015, for which we extracted meteorological exposure data for the three trimesters preceding each birth. We used linear regression, controlling for key covariates, to estimate the timing, strength, and direction of meteorological effects on birth weight. Our results indicated that precipitation during the third trimester had a positive association with birth weight, with more frequent days of precipitation associated with higher birth weight: we observed a 3.1g (95% CI: 1.0–5.3g) increase in birth weight per additional day of exposure to rainfall over 5mm. Increases in average daily temperature during the third trimester were also associated with birth weight, with an increase of 41.8g (95% CI: 0.6–82.9g) per additional degree Celsius. When the sample was stratified by season of birth, only infants born between June and November experienced a significant associated between meteorological exposures and birth weight. The association of meteorological variation with foetal growth seemed to differ by ethnicity; effect sizes of meteorological were greater among an Indigenous subset of the population, in particular for variation in temperature. Effects in all populations in this study are higher than estimates of the African continental average, highlighting the heterogeneity in the vulnerability of infant health to meteorological variation in different contexts. Our results indicate that while there is an association between meteorological variation and birth weight, the magnitude of these associations may vary across ethnic groups with differential socioeconomic resources, with implications for interventions to reduce these gradients and offset the health impacts predicted under climate change.

## 1. Introduction

Climate change disproportionately impacts health for those already facing the greatest burden of ill health and social inequality [1, 2]. Adverse impacts are highest among poor populations reliant on subsistence farming and with low access to health services [3]. In this context, rural and remote populations in sub-Saharan Africa and Indigenous populations worldwide are among the most vulnerable globally. Research on climate-sensitive health outcomes has primarily focused on the more direct associations between climate or meteorology and health, examining the effects of climate or weather on vector-borne diseases such as malaria, waterborne diseases such as cholera, food security and nutrition, or on the health impacts of extreme weather events [1, 4–6]. However, the bulk of climate impacts on health are expected to come from more distal, indirect, and diverse impacts on health outcomes not typically or widely recognized as climate sensitive [7–9].

There has been increasing interest and research into the extent to which meteorological variation impacts foetal development [10–13]. The majority of studies investigating weather impacts on birth outcomes have taken place in high-income countries, where the mechanisms and context through which meteorological factors influence foetal growth may differ compared to developing regions [13, 14]. Many of the known determinants for low birth weight in developing countries are seasonally patterned, among them major predictors of birth weight: maternal energy intake during pregnancy, pre-pregnancy body mass index (BMI), weight gain during pregnancy [15]. According to Molina and Saldarriaga [16], there are five pathways through which temperature can affect the health of a developing foetus: exposure to extreme temperatures, maternal infection by a temperature-sensitive disease (e.g., respiratory infections) or by a biological vector-borne disease, maternal mental illnesses, and food insecurity brought about by less predictable growing conditions. Similarly, rainfall has been posited to affect foetal health through the reduced crop yields in the dry season that may result in maternal nutritional deficits at these times. Nevertheless, few empirical studies have explored the relationship between seasonal or meteorological effects and birth weight at the regional level and in a sub-Saharan African context. Grace, Davenport [17] address this research gap in their examination of data from 19 countries across Africa. Their results indicate that maternal exposure to high temperatures during gestation had a negative effect on birth weight, while exposure to increased levels of precipitation during early pregnancy had a positive effect in some settings. The latter finding is surprising as it differs from those of the seminal Dutch Famine study on the timing of nutritional insults in utero and foetal birth weight [18]. Given substantial variation in regional climates and birthing practices across Africa, however, it remains unclear whether weather homogenously impacts birth outcomes across countries and in diverse contexts. We anticipate that at more regional scales, the nature of associations between meteorological exposures and birth weight may differ in both direction and magnitude and that characterizing these individual patterns is essential to climate change adaptation interventions tailored to specific circumstances.

We contribute to the small but emerging evidence base examining meteorological associations with birth outcomes in vulnerable populations in sub-Saharan Africa. More specifically, we evaluate the association between meteorological factors and foetal growth in a sample of births from Bwindi Community Hospital in Kanungu District, Uganda. We hypothesize that meteorological drivers will be associated with foetal growth, that these effects will be greatest in the third trimester (in accordance with the theory of third trimester nutritional deprivation having the effect on birth weight [18]), and that effects will modified by ethnicity (differing between Indigenous and non-Indigenous mothers). Specific objectives included: 1) assessing the impact of meteorological variation on birth weight, 2) identifying the highest-risk period for meteorological exposures during pregnancy, and 3) comparing the effects of meteorological exposures on birth weight in Indigenous and non-Indigenous mothers inhabiting the same region.

### 1.2 Determinants of size at birth

Size at birth is a function of the length of gestation and the rate of growth in utero [19]. Birth weight correlates with gestation length [20], and is thus not an independent measure of a baby’s health status, though it is often the only available measure in low-resource settings [21]. More appropriate measures consider weight in relation to gestation and provide classifications (small for gestational age [SGA], appropriate for gestational age [AGA]) according to gestational age-specific percentiles, which can be used as a proxy of intrauterine growth restriction [IUGR]). The rate of foetal growth can vary depending on various factors, one of the most important being based on the timing of any nutritional insults to which the foetus is exposed. Results from the influential Dutch famine study indicated that nutritional deprivation during the third trimester in particular can have a detrimental effect on birth weight [18, 22].

The aetiologic determinants of IUGR differ from those of preterm birth [15]. In the developing country context, the following have been identified as key determinants of IUGR (in decreasing order of importance): low energy intake/gestational weight gain, low pre-pregnancy BMI, short stature, malaria (for primiparae in malaria-endemic areas), cigarette smoking (where maternal smoking during pregnancy is prevalent [10–20%]), primiparity, pregnancy-induced hypertension, congenital anomalies; and other genetic factors [15]. As with many health outcomes, low birth weight is patterned by socioeconomic disparities, though the majority of studies on these patterns have been undertaken in developed country settings [23–27].

## 2. Methods

### 2.1 Study location and population

**Fig 1:**
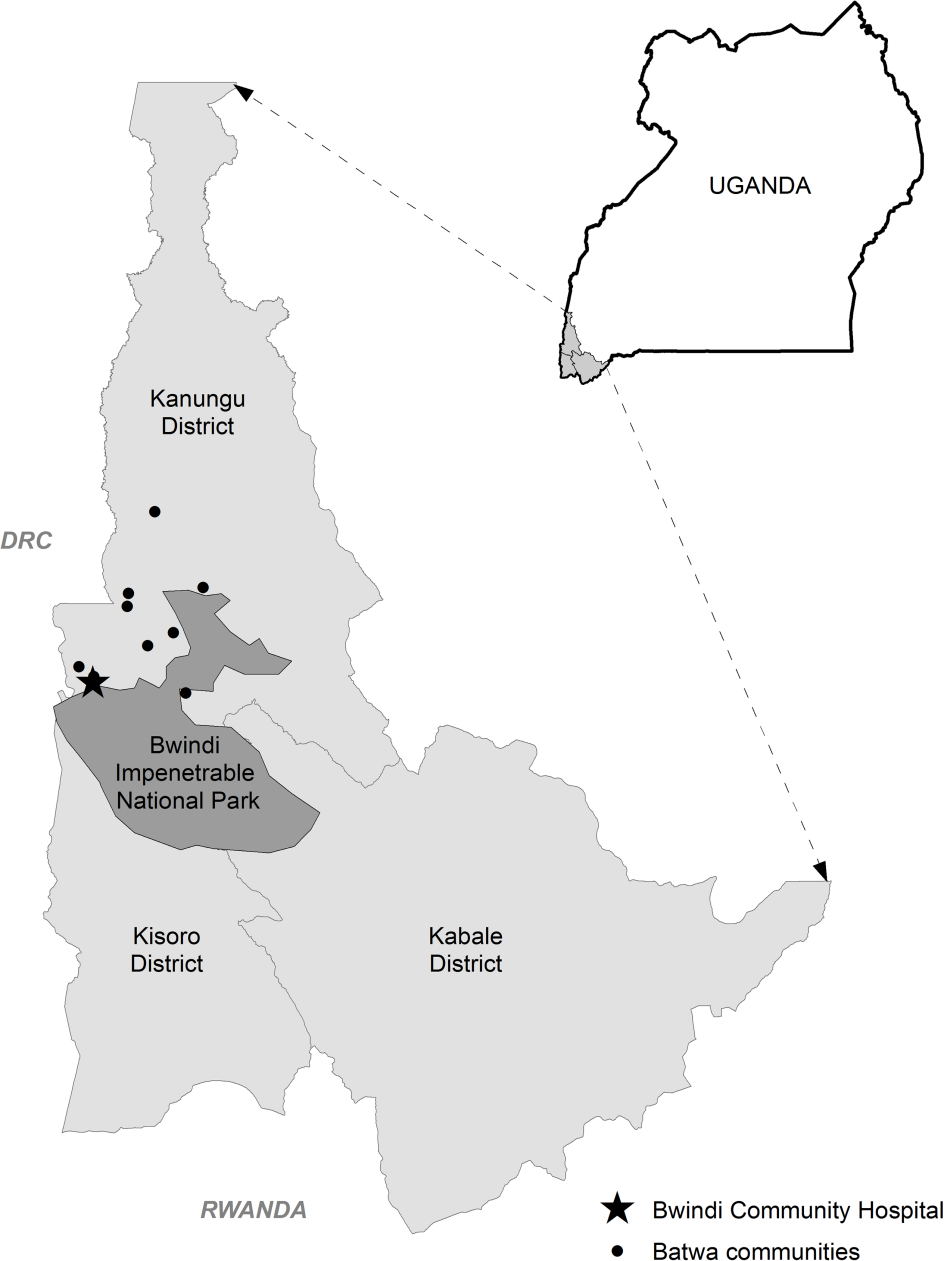
Map of the study region, Kanungu District, Uganda. The study area is located northwest of Bwindi Impenetrable National Park, hemmed in by the border of the Democratic Republic of the Congo (DRC).

Our study sample consists of 3691 women who gave birth at Bwindi Community Hospital in Kanungu District, Uganda, between June 2012 and June 2015. Kanungu District is situated in southwestern Uganda, near the borders of the Democratic Republic of the Congo and Rwanda (Fig 1). The study area is situated in the region surrounding Bwindi Impenetrable National Park, which is inhabited predominantly by the Bakiga ethnic group as well as approximately 900 members (1%) of the Indigenous Batwa population [28, 29]. Both the Bakiga and Batwa have high burden of ill health relative to the national average, with particularly high inequality and burden of ill health among the Indigenous Batwa [28]. Evicted from their forest homes when the National Park was created in the early 1990s, the Batwa were forced to resettle in agrarian communities [28, 30, 31]. The existing burden of ill health among the Batwa has been characterized across a variety of metrics, including reduced life expectancy (28 years for the Batwa compared to the Ugandan average of 53 [28]), higher prevalence of malaria (9.4% for the Batwa compared to 4.5% in the Bakiga population [32]), acute gastrointestinal illness (compared to East Africa [33]), and extreme food insecurity [34]. The prevalence of HIV among the Batwa population is, however, lower than that in the Bakiga population [35]. Community-based health surveys in Kanungu District (in the same communities from which our hospital sample was derived) have established a significant correlation between ethnicity and indicators of socio-economic status (SES) (Table 1). Donnelly, Berrang-Ford [32] found significantly lower levels of education and asset ownership among Indigenous Batwa compared to the non-Indigenous population. Donnelly, Berrang-Ford [32] also show that ethnicity and SES had both independent and collinear effects on malaria infection, implying that ethnicity may act as a partial proxy for gradients in SES in this population. There is also a higher burden of malnutrition among Batwa women than among Bakiga women [36].

**Table 1:** Indicators of socioeconomic status among Batwa and Bakiga communities.

Measure (variable descriptor)	Batwa (proportion of the population)	Bakiga (proportion of the sample)	Source
Malaria prevalence among adults (positive malaria antigen detection test)*	29 (6.45)	20 (4.46)	[32]
Moderate acute malnutrition among adult women (classified as moderately malnourished according to Uganda Ministry of Health Integrated Management of Acute Malnutrition Guidelines)	61 (45.86)	1 (0.42)	[36]
Household mosquito net use (did not have nets)	93 (70.99)	218 (53.56)	[32] (by request)
Assets (did not have any assets)	82 (62.12)	77 (19.01)	[32] (by request)
Access to handwashing facilities (did not have access to handwashing)	96 (73.85)	229 (56.40)	[32] (by request)
Access to soap (did not have access to soap)**	98 (75.38)	252 (62.06)	[32] (by request)

*Prevalence in July 2013 and April 2014—survey of all Batwa adults, sample of Bakiga adults

**Only asked of people that had access to hand washing facility, for example for the Batwa, 32 or 94% of the households that had access to handwashing had access to soap

Research has documented high vulnerability to the health impacts of climate change within both the Batwa and Bakiga populations, and both groups have identified malaria, food insecurity, and gastrointestinal illnesses as climate-sensitive health concerns [28, 29]. Lack of access to health care and the strenuous nature of subsistence farming labour also give rise to substantial disparities in perinatal health in Kanungu District. Approximately 40% of births in the region occur in health facilities [37] compared to 57% country-wide [38]. This disparity is paralleled by similar metrics examining the presence of a skilled healthcare provider at delivery: 59% of all Ugandan infants are delivered by a skilled provider, compared to 42% of infants in the Southwest Region [37].

Bwindi Community Hospital (BCH) was established in 2003 as an outreach clinic for the Batwa, but has expanded into a full inpatient hospital serving 100 000 people across three sub-counties in Kanungu district [39]. The hospital is located in Buhoma trading centre, and also operates several satellite clinics in more remote settlements. The hospital’s antenatal clinic sees approximately 250 mothers per month and performs over 1000 deliveries each year [40]. BCH also provides antenatal care and family planning services. In 2008, BCH opened a ‘Waiting Mothers Hostel,’ where women who live at greater distances from the hospital can stay while they are waiting to give birth [41].

The climate in Kanungu includes two rainy seasons: the ‘short rains’ from October to December and the ‘long rains’ from March to May [42]. Average temperatures in the region are relatively cool (typically below 20°C) in comparison to the rest of the country, though there has been an increase in mean annual temperature of 1.3°C over the last fifty years [42]. Global climate models for the region predict increases in both mean annual temperature and heavy rain events [43, 44] along with increases in severe dry conditions in August and September [45] and trends of increasing drought [46]. Southwestern Uganda has been reported as the fastest warming region in the country [47]. Dominant livelihood activities in the region include agriculture, industrial tea and coffee production, and tourism [28, 29].

### 2.2 Birth outcome measures

Birth outcomes were ascertained from the hospital records that were completed by nurses during labour and after birth. They include information on the mother’s medical history, intranatal measures and interventions, and assessments of the baby at birth. Birth weight in grams is our primary outcome. In the available birth record data from Bwindi Community Hospital (2012–2015), there were 3691 births, 3343 of which were singleton births. Of these, there were 3197 complete records with sufficient information to estimate gestational age.

Gestational age (GA) was estimated for all singleton birth records (n = 3197) to determine the exposure windows of the meteorological variables and to include as a key control variable in our ‘base’ regression models for the primary outcome analysis. In the case of 591 observations (17.9%), gestational age was recorded based on ultrasound dating, the most reliable measurement standard for GA. The date of the ultrasounds were not collected, but we note that ultrasounds conducted later in pregnancy have a greater margin of error than those conducted within the first trimester, and that there is variation in the timing of initial presentation of pregnant women to the hospital or antenatal clinic. Where GA based on ultrasound was not available, last menstrual period (LMP, determined by maternal recall, n = 1742, 52.7%) and adjusted fundal height (n = 971, 29.4%) were used. Fundal height measures (estimated by midwives by hand measurements, typically without a measuring tape) upon presentation for delivery were compared with ultrasound expected delivery dates and adjusted to control for fundal height underestimation of gestation length by an average of two weeks. If the gestational age based on LMP exceeded 330 days [48], fundal height was used to estimate GA.

### 2.3 Meteorological data

Meteorological predictor selection was based on the literature and variation in weather in the study area. We selected the number of days of precipitation (as per Grace et al. (2015)) above 5mm during the exposure window (described below) as our meteorological exposure for the effects of precipitation in our models. Given the limited range of temperature fluctuations in the region, the mean of daily temperature (°C) during each exposure period was selected to examine the effects of temperature on the outcome variables. Birth season was included in all models as a fixed effect to control for any unaccounted effects of seasonality [49].

Meteorological data for rainfall and temperature were extracted and matched to each birth for multiple exposure periods reflecting the entire pregnancy period and each trimester. Daily rainfall data were estimated based on satellite observations using the Rainfall Estimator, version 2.0 (RFE2) algorithm [50, 51]. In this algorithm, rainfall amounts estimated from geostationary satellite infrared images at high spatial and temporal resolutions are calibrated against ground rain gauge data as well as satellite microwave measurements. These data were then interpolated to the Buhoma region to form a daily rainfall time series for the study period. We processed daily temperature data from the ERA-Interim (ERAi) reanalysis dataset of European Center of Medium-range Weather Forecasts [52]. This dataset is generated by assimilating meteorological measurements from various observational sources into a global numerical model and then forecast at high temporal resolution. We interpolated gridded 3-hourly ERAi temperature data at 0.75 degree resolution to Buhoma site. The daily mean/maximum/minimum temperatures were then analyzed from the 3-hourly temperature time series at the site. We validated our extracted daily rainfall and temperature data against local meteorological measurements (approximately 12 months) for a weather station in Buhoma Town. Both rainfall and temperature variables were highly correlated with the station measurements at higher than a 99% confidence level.

### 2.4 Covariates

We controlled for variables known to influence birth weight (Table 2). We included infant sex as a control variable as male infants are typically larger than female infants [53]. To estimate effects of meteorological exposures on foetal growth in utero, we controlled for gestational age. Though foetal growth is approximated as gestational age-specific birth weight, we included a categorized gestational age as a covariate to reduce measurement errors due to heterogeneous measures of gestational age in the study sample. Based on available data and known determinants of birth weight [54], we included maternal age, ethnicity, parity, and maternal marital status as control variables. HIV status was also included as a control as infants born to HIV-positive mothers are more likely to be classified as low birth weight (LBW) [55]. We considered delivery type as a possible control variable; while delivery type would not be a determinant of LBW, it can be an indicator of complications, and was thus included in models. There were three classifications for delivery type: spontaneous vaginal birth, assisted vaginal birth (i.e. via vacuum extraction or with forceps), or Caesarean section. Whether or not a woman had an ultrasound scan during her pregnancy was included as a proxy for access and quality of antenatal care, as only women who attended antenatal care underwent ultrasounds and ultrasounds were generally only available in the better-equipped hospital-based clinics (as opposed to satellite clinics in more remote areas). Women who attended antenatal care at a facility with ultrasound services are meant to undergo two scans at different points in their pregnancy, but often undergo only one. The timing of the first scan is dependent upon the date of the first antenatal care visit, which occurs in the second trimester for the majority of women attending antenatal care at Bwindi Community Hospital [56]. The ultrasound costs 1000 Ugandan shillings, a cost that the BCH sonographer expressed may be prohibitive to some mothers with limited financial means [57]. Women experiencing complications or who are primigravida may undergo ultrasound scans sooner and/or more often than women whose pregnancies are progressing normally [56].

**Table 2:** Table of variables used in regression analyses.

**Variable (Units)**	**Description**
***Dependent (outcome) variable***	
Birth weight (g)	Continuous
***Independent (predictor) variables***	
Mean of average daily temperature values (°C) over the exposure period	Continuous
Number of days with precipitation >5 mm during exposure period	Count based on dichotomous condition for each day of pregnancy: rainfall <5mm = 0, rainfall >5mm = 1
***Control variables***	
Infant sex	Dichotomous: 0 = male, 1 = female
Gestational age	Categories: 0 = full term (≥259 days), 1 = moderate to late preterm (224–259 days), 2 = very preterm (196–224 days), 3 = extremely preterm (<196 days)
Ethnicity	Categories: 0 = Bakiga, 1 = Batwa, 3 = Other
Maternal age	Continuous
Maternal marital status	Dichotomous: 0 = unmarried, 1 = married
Maternal parity	Number of prior pregnancies to delivery
Delivery type	Categories: 0 = spontaneous vaginal delivery, 1 = assisted vaginal delivery, 2 = Caesarean
Maternal HIV status	Dichotomous: 0 = HIV-, 1 = HIV+
Maternal malaria	Dichotomous: 0 = no malaria during pregnancy, 1 = malaria during pregnancy
Season of birth	Categories: 1 = Dec-Feb (dry), 2 = Mar-May (rain), 3 = Jun-Aug (dry), 4 = Sep-Nov (rain)
Ultrasound	Dichotomous: 0 = no ultrasound, 1 = ultrasound

We included ethnicity to compare Indigenous vs. non-Indigenous birth outcomes in the population, which in this context serves as a partial proxy for socio-economic status (see Section 2.1). We hypothesized that ethnicity would act not only as a control variable, but also an effect modifier for meteorological effects on birth weight. This hypothesis is consistent with climate change adaptation literature, which theorizes that climatic and/or meteorological effects will manifest through, and be modified by, existing gradients in health [2, 4, 6, 7]. Our ethnicity variable had low variation; the small subset of Indigenous Batwa limited the statistical power of analyses using data from this subset.

### 2.5 Data analysis

Our analytical sample included live singleton births with information on birth weight, gestational age, and all known covariates (n = 3197). Linear regression was used to estimate mean differences in birth weight associated with meteorological exposures. We constructed a ‘base’ model that includes infant sex and gestational age to examine sex- and gestational age-specific birth weight differences by exposure and potential confounders. Gestational age was included as a categorical control variable due to the potential relatively high measurement errors inherent to LMP- and fundal height-based measurements and a non-linear relation to birth weight suggested by the lowess-smoothed scatterplot.

We then built models that included all control variables and meteorological exposures, constructing four models: a model with meteorological exposures and all control variables for each of the three trimesters, and a fourth model with meteorological exposures and all control variables for the entire pregnancy period. We stratified the sample by ethnicity and tested the same models in each group to investigate ethnicity as an effect modifier. In a separate analysis, we stratified the sample by season of birth and tested the models for each exposure period.

We conducted multiple sensitivity analyses to examine robustness of associations: (1) re-analyzing the sample after excluding preterm births (<37 weeks), (2) re-analyzing the sample after excluding all cases for which GA was not determined by ultrasound, (3) examining associations using different thresholds for temperature (e.g., maximum and minimum temperature exposures) and precipitation (e.g., exposure to number of days with rainfall over 1mm, or number of days with rainfall over 10mm), (4) testing for interaction effects between meteorological exposures and infant sex, and (5) testing models with month of conception instead of season of birth to evaluate any non-random seasonal fertility selection. Further sensitivity analyses evaluated LBW as an outcome using logistic regression. This analysis allowed us to examine the lower 5–10% of birth weights separately, since as Grace, Davenport [17] state, this lowest distribution of birth weights is not accurately captured as a continuous dependent variable.

To assess model fit, we evaluated collinearity by examining the Pearson correlation coefficient matrix for all predictor variables and control variables. We conducted post-estimation tests to assess heteroscedasticity (Breysch-Pagan test) and the normality of the residuals (Q-Q plots and Shapiro-Wilk tests) in our models. We checked that the outcome variable was normally distributed (in both the overall samples and those stratified by ethnicity). We also evaluated the linearity of the meteorological exposure variables and other control variables using scatterplots, as well as lowess smoothing to visually assess trends in these plots. Data were analysed using Stata v.13 (StataCorp, USA).

### 2.6 Ethics

This study was approved by the McGill University and the University of Guelph Research Ethics Boards (REB File #461–0414). Additionally, the research team has a Memorandum of Understanding with, and received approval for this study from, Bwindi Community Hospital (BCH). The study design conforms to the Canadian Tri-council Policies and follows the requirements of the Ethical Conduct of Research Involving Human Subjects; it is also in compliance with Ugandan laws and regulations for foreign researchers. As per McGill, Tri-Council, and BCH ethical research policies, informed consent by individual patients specific to this study was not required. This study included retrospective analysis of de-identified hospital records, and consent to use the data for hospital and hospital-approved research analysis is provided by the patient at the time of admission to the hospital or maternity ward.

## 3. Results

### 3.1 Descriptive results

The average age of mothers in the study was 24.66 (SD: 5.76) years old, with a mean of 2.72 previous pregnancies (SD: 2.09) and a mean parity of 1.63 (SD: 1.86) births per woman (Table 3). The majority of mothers (87.5%) were of Bakiga ethnicity, with a minority of Batwa Indigenous ethnicity (1.0%) or other non-Indigenous ethnic groups (11.5%). The prevalence of HIV in the sample was 9.2%, and 34 (1.0%) mothers reported falling ill with malaria during pregnancy. Nearly 18% of mothers had an ultrasound during their pregnancy. The proportion of babies delivered by C-section (31.8%) was higher than the national average (5%, UNICEF [38]). This higher proportion of C-section deliveries reflects the fact that the births examined in this study all occurred in a hospital setting, compared to national statistical inclusion of all births. BCH is also a large referral hospital receiving high-risk birth events from smaller referral clinics. Mean birth weight was 3087.99g (SD: 482.93g), with 237 births classified as LBW (7.2%). The mean gestation length was 278.03 (SD: 16.51) days, and 256 (7.75%) of the births were preterm. Over the course of pregnancy, the mean temperature exposure was 19.41°C (SD: 0.31) and the mean number of days of precipitation >5mm experienced was 61.60 (SD: 12.49) days.

**Table 3:** Descriptive summary of birth outcome, meteorological and control variables in the dataset.

		**N. (%)**	**Estimate (SD)**
Meteorological variables			
Season of birth (n = 3304)			
	*Dec-Feb dry*	835 (25.3)	
	*Mar-May rainy*	801 (24.2)	
	*Jun-Aug dry*	791 (23.9)	
	*Sep-Nov rainy*	877 (26.5)	
Average number of days of rain >5mm during pregnancy (n = 3124)			61.60 (12.49)
Average temperature (°C) during pregnancy (n = 3124)			19.41 (0.31)
Maternal variables			
Average age of mothers (n = 3265)			24.66 (5.76)
	*<20*	1122 (31.0)	
	*20–24*	808 (22.3)	
	*25–29*	389 (10.7)	
	*30–34*	624 (17.2)	
	*≥35*	681 (18.8)	
Average number of pregnancies including current (n = 3246)			2.72 (2.09)
Average parity (n = 3261)			1.63 (1.86)
Maternal marital status (n = 3269)			
	*Married*	3062 (93.7)	
	*Unmarried*	207 (6.3)	
HIV status (n = 3205)			
	*Negative*	2910 (90.8)	
	*Positive*	295 (9.2)	
N. with malaria during pregnancy (n = 3274)		34 (1.0)	
Ethnicity (n = 3304)			
	*Bakiga*	2890 (87.5)	
	*Batwa*	33 (1.0)	
	*Other non-Indigenous*	381 (11.5)	
Antenatal care and delivery variables			
Ultrasound (n = 3304)			
	*Had ultrasound*	591 (17.9)	
	*Did not have ultrasound*	2713 (82.1)	
Delivery type (n = 3275)			
	*Spontaneous vaginal birth*	2119 (64.7)	
	*Assisted vaginal birth*	116 (3.5)	
	*Caesarean section*	1040 (31.8)	
Infant variables			
Infant sex (n = 3294)			
	*Male*	1679 (51.0)	
	*Female*	1615 (49.0)	
Average gestation length (days) (n = 3197)			278.03 (16.51)
	*Full term (≥259 days)*	2939 (91.9)	
	*Moderate to late preterm (224–259 days)*	218 (6.8)	
	*Very preterm (224–196 days)*	26 (0.8)	
	*Extremely preterm (<196 days)*	14 (0.4)	
Birth weight (n = 3304)			3087.99 (482.93)
N. of low birth weight cases (<2500g) (n = 3304)		237 (7.2)	

### 3.2 Associations between study variables and birth weight

Birth weight increased significantly with maternal age, among married mothers, in babies born via C-section, and with increasing parity after controlling for gestation age and infant sex (Table 4). The results for maternal age, maternal marital status, delivery type, parity, and undergoing ultrasound scan were significant and consistent across models examining the full sample and those stratified by ethnicity. Infant sex had a substantial effect on birth weight; being born female corresponded to an approximately 114.5g decrease in birth weight (95% CI: -147.1 –-81.8g). Babies born to Indigenous Batwa mothers had lower birth weights (-297.4g, 95% CI: -456.8 –-138.0g) compared to those born to Bakiga mothers. Season of birth was not a significant predictor of birth weight. Estimates in the Batwa sub-sample had wide confidence intervals, reflecting the small sample size for this subset. Despite the limited statistical power of the Batwa subset, however, gestational age, most categories of maternal age, whether or not a mother had an ultrasound, and HIV status were significant exposures for birth weight. We note however, that there were no births classified as very preterm or extremely preterm in the Batwa sample, effectively making gestational age a dichotomous variable in this sample.

**Table 4:** Associations between study variables and birth weight (all models control for infant sex and gestational age).

	Full sample (n = 3197)		Batwa subset (n = 32)	
Variable	Coefficient (g)	95% Confidence Interval	Coefficient (g)	Confidence Interval (95%)
**Independent (predictor) variables**				
Season of birth (ref = Dec-Feb dry)				
*Mar-May rainy*	-9.94	-55.21–35.33	5.03	-536.80–546.85
*June-Aug dry*	-5.97	-51.45–39.51	73.50	-487.01–634.01
*Sep-Nov rainy*	-27.85	-72.16–16.45	-119.01	-589.36–351.35
Mean temperature (°C)				
*Trimester 1*	-11.58	-40.76–17.60	270.09	-208.21–748.39
*Trimester 2*	1.58	-30.68–33.79	114.59	-154.77–383.95
*Trimester 3*	15.22	-12.50–42.94	199.61	-71.42–470.63
*Entire pregnancy*	3.87	-49.60–57.34	587.81*	57.39–1118.24
Number of days with rainfall >5mm				
*Trimester 1*	0.52	-1.42–2.47	-5.58	-26.29–15.13
*Trimester 2*	0.25	-1.61–2.11	-0.04	-17.44–17.37
*Trimester 3*	1.74*	0.04–3.45	-1.32	-21.44–18.80
*Entire pregnancy*	1.64*	0.31–2.96	-3.28	-17.44–10.88
**Control variables**				
Infant sex (ref = male)	-114.48***	-147.11 –-81.84	-18.38	-300.52–337.28
Gestational age (ref = full term, ≥259 days)				
*Moderate to late preterm (224–259 days)*	-271.38***	-335.46 –-207.29	-550.27*	-1080.01 –-20.53
*Very preterm (224–196 days)*	-778.96***	-962.17 –-595.75		
*Extremely preterm (<196 days)*	-871.98***	-1147.42 –-596.53		
Maternal age (ref = <20)				
*20–24*	82.77***	41.35–124.18	**-295.14†**	-617.99–27.70
*25–29*	69.65*	16.71–122.60	-274.69	-685.70–136.33
*30–34*	116.51***	58.75–174.27	-852.23***	-1253.71 –-450.75
*≥35*	-162.39***	-205.99 –-118.80	-642.63***	-970.51 –-314.76
Parity	38.05***	29.50–46.60	-25.10	-104.47–54.27
Marital status (ref = unmarried)	164.35***	98.82–229.87	-64.54	-475.49–346.41
Ethnicity (ref = Bakiga)				
*Batwa*	-297.36***	-456.75–-137.97		
*Other*	-7.74	-57.70–42.23		
Ultrasound (ref = no)	46.28*	4.69–87.87	453.23**	145.19–761.28
Delivery type (ref = spontaneous vaginal delivery)				
*Assisted vaginal delivery*	-34.10	-120.51–55.31	-193.39	-844.37–457.58
*Caesarean section*	108.81***	74.32–143.30	265.19	-74.87–605.24
HIV status (ref = HIV-negative)	-34.66	-90.23–20.91	695.54**	215.14–1175.94
Malaria in pregnancy (ref = no malaria during pregnancy)	-105.43	-265.24–54.38	-	-

***p<0.001

**p<0.01

*p<0.05

†p<0.10

### 3.3 Meteorological predictors of birth weight

In models testing crude associations of exposure variables with birth weight (adjusting for gestational age and infant sex only) (Table 4), exposure to precipitation in the third trimester and throughout pregnancy was associated with a significant but modest increase in birth weight. Though temperature exposure was not significantly associated with birth weight in models testing its effect in the full sample or the non-Indigenous subset, mean temperature exposure over the course of entire pregnancy was borderline significant predictor of birth weight in the Batwa subset (p = 0.06).

Multivariable models tested the effects of meteorological predictors on birth weight adjusting for all covariates in Table 4 (Table 5). Precipitation exposure in the third trimester and over the course of the entire pregnancy were positively associated with birth weight. Exposure to an additional day of precipitation >5mm during the third trimester corresponded to a 3.1 g (95% CI: 1.0–5.3g) increase in birth weight. In other words, each additional week of exposure to daily rainfall >5mm in the third trimester corresponds to an increase in birth weight of approximately 21.7g, which is comparable to the effect of parity (17.9g, 95% CI: 5.2–30.6g) on birth weight in the study sample. The coefficient for temperature exposure in the third trimester model indicated that for each degree increase in mean temperature exposure, birth weight increased by 41.8g, though the confidence interval was wide (95% CI: 0.6–82.9g). The direction and magnitude of effects of all control variables (shown in Table 4 but not in Table 5) did not differ substantially across models or from a baseline model that did not contain meteorological predictor variables.

**Table 5:** Trimester-specific associations between meteorological exposures and birth weight, stratified by ethnicity.

		All cases		Batwa	
Trimester	Meteorological Variables	Coefficient	Confidence Interval (95%)	Coefficient	Confidence Interval (95%)
*Trimester 1*	Number of days with rainfall >5mm	-0.11	-2.58–2.38	1.40	-23.74–26.55
	Mean temperature (°C)	-20.68	-69.41–28.05	253.87	-487.19–994.93
*Trimester 2*	Number of days with rainfall >5mm	-0.39	-3.08–2.29	0.41	-31.34–32.16
	Mean temperature (°C)	3.31	-48.07–54.70	245.05	-275.45–765.54
*Trimester 3*	Number of days with rainfall >5mm	3.12**	0.97–5.27	9.75	-19.60–39.10
	Mean temperature (°C)	41.78*	0.64–82.92	**405.07†**	-24.56–834.69
*Entire pregnancy*	Number of days with rainfall >5mm	1.63*	0.06–3.21	3.00	-15.23–21.24
	Mean temperature (°C)	26.03	-35.33–87.40	571.23	-141.70–1284.17

***p<0.001

**p<0.01

*p<0.05

†p<0.10

*N*.*B*. Models include all control variables from the baseline model (Table 4). Coefficients and confidence intervals for control variables are not shown, but did not substantively differ from the baseline model. Each model includes both rainfall and temperature variables together in the model. Models are stratified by trimesters and population subsets.

Strikingly, given the small sample size of the Batwa subset, mean temperature exposure during the entire pregnancy was positively associated with birth weight in the Indigenous (Batwa) subset (Table 4). Each degree increase in mean temperature exposure throughout pregnancy corresponded to an increase of 587.8g (95% CI: 57.4–1118.2g). In the multivariable models, there was some indication that third trimester mean temperature exposure corresponded to an increase of 405.1g (95% CI: -24.6–834.7g) in birth weight (Table 5). The effect sizes of precipitation on birth weight among Batwa were approximately 2–3 times greater than among the non-Indigenous population, and these estimates fell outside of the confidence intervals for the non-Indigenous population for individual trimesters (but not the entire pregnancy), and were not independently significant in the Batwa subset model.

In addition to this association observed between birth weight and temperature exposure during the third trimester, sensitivity analyses of models for the Batwa subset with an adjusted precipitation variable with a lower threshold (inclusive of days with rainfall over 1mm) and of models restricted to term births (≥37 weeks) indicated that there may also be a significant association between mean temperature exposure incurred throughout pregnancy and birth weight.

The trends we observed in Table 4 for all cases led to further inquiry to test if the patterns of association might differ by season of birth (Table 6). In models stratified by season of birth, we observed no significant associations between meteorological exposures and birth weight for births occurring in seasons 1 and 2 (December–February dry season and March–May rainy season, respectively). However, in the driest season, season 3 (June–August), each degree increase in average temperature exposure during the third trimester corresponded to a 123.1g (95% CI: 19.0g – 227.2g) increase in birth weight. There were also significant associations between birth weight and precipitation exposures for babies born between June and August. For the births in this period, increased exposure to days of rainfall >5mm in the second trimester was associated with a -6.2g (95% CI: -11.8g –-0.7g) decrease in birth weight and exposure in the third trimester with a 9.4g (95% CI: 4.3g – 14.5g) increase in birth weight. Overall, each additional day of exposure to rainfall >5mm throughout pregnancy was associated with a 4.8g (95% CI: 1.6g – 8.0g) increase in birth weight. For infants born in season 4 (September–November, wet period), there was only a significant association between mean temperature exposure in the third trimester and birth weight—a 98.4g (95% CI: 23.0g – 173.7g) increase for each additional degree of average temperature exposure.

**Table 6:** Trimester-specific associations between meteorological exposures and birth weight, stratified by season of birth.

Trimester	Meteorological Variables	Coefficient	Confidence Interval (95%)
**Season 1 (December–February *DRY*)**			
*Trimester 1*	Number of days with rainfall >5mm	1.35	-4.16–6.87
	Mean temperature (°C)	-40.38	-152.67–71.92
*Trimester 2*	Number of days with rainfall >5mm	-1.72	-6.90–3.45
	Mean temperature (°C)	9.85	-78.62–98.31
*Trimester 3*	Number of days with rainfall >5mm	1.24	-2.67–5.15
	Mean temperature (°C)	-4.72	-83.86–74.41
*Entire pregnancy*	Number of days with rainfall >5mm	0.40	-3.43–4.23
	Mean temperature (°C)	-23.85	-132.13–84.43
**Season 2 (March–May *RAINY*)**			
*Trimester 1*	Number of days with rainfall >5mm	-1.85	-7.52–3.83
	Mean temperature (°C)	-7.82	-100.74–85.09
*Trimester 2*	Number of days with rainfall >5mm	1.78	-3.65–7.21
	Mean temperature (°C)	53.24	-47.50–153.99
*Trimester 3*	Number of days with rainfall >5mm	2.94	-2.30–8.19
	Mean temperature (°C)	-49.11	-133.86–35.65
*Entire pregnancy*	Number of days with rainfall >5mm	0.57	-2.65–3.80
	Mean temperature (°C)	24.95	-89.68–139.57
**Season 3 (June–August *DRY*)**			
*Trimester 1*	Number of days with rainfall >5mm	4.05	-1.35–9.45
	Mean temperature (°C)	34.80	-65.99–135.60
*Trimester 2*	Number of days with rainfall >5mm	-6.24*	**-11.76 –-0.72**
	Mean temperature (°C)	-58.89	-212.87–95.08
*Trimester 3*	Number of days with rainfall >5mm	9.43***	**4.33–14.52**
	Mean temperature (°C)	123.06*	**18.95–227.18**
*Entire pregnancy*	Number of days with rainfall >5mm	4.81**	**1.58–8.03**
**Season 4 (September–November *RAINY*)**			
*Trimester 1*	Number of days with rainfall >5mm	-1.66	-6.20–2.88
	Mean temperature (°C)	**-131.64†**	**-265.69–2.41**
*Trimester 2*	Number of days with rainfall >5mm	4.75	-1.41–10.91
	Mean temperature (°C)	12.67	-105.68–131.02
*Trimester 3*	Number of days with rainfall >5mm	3.17	-0.78–7.11
	Mean temperature (°C)	98.37*	**23.04–173.70**
*Entire pregnancy*	Number of days with rainfall >5mm	1.29	-1.64–4.22
	Mean temperature (°C)	-3.18	-133.95–127.58

***p<0.001

**p<0.01

*p<0.05

†p<0.10

*N*.*B*. Models include all control variables from the baseline model (Table 4). Coefficients and confidence intervals for control variables are not shown, but did not substantively differ from the baseline model. Each model includes both rainfall and temperature variables together in the model. Models are stratified by trimesters.

Results of our sensitivity analyses of subsets restricted to term births and cases with ultrasound GA measures showed consistent results in both direction and statistical significance of associations (Table 7). Models without a control variable for gestational age also showed associations consistent with those presented in Table 5. We also observed that results were robust across varying temperature and precipitation thresholds for models examining both the full sample and the Indigenous and non-Indigenous subsets. However, results of models with a dichotomous outcome variable (LBW) did not indicate that meteorological variables were significant predictors of LBW classification.

**Table 7:** Sensitivity analyses of multivariable models for subsets of term births and ultrasound-dated births.

		Term births		Ultrasound-dated births	
Trimester	Meteorological Variables	Coefficient	Confidence Interval (95%)	Coefficient	Confidence Interval (95%)
*Trimester 1*	Number of days with rainfall >5mm	0.46	-2.06–2.97	-1.30	-7.58–4.99
	Mean temperature (°C)	-14.42	-64.33–35.50	-13.46	-139.12–112.20
*Trimester 2*	Number of days with rainfall >5mm	0.32	-2.39–3.03	3.91	-2.66–10.49
	Mean temperature (°C)	14.19	-38.21–66.59	38.84	-90.90–168.58
*Trimester 3*	Number of days with rainfall >5mm	3.92**	1.55–6.30	10.59**	3.47–17.72
	Mean temperature (°C)	48.14**	5.21–91.07	147.58*	25.14–270.03
*Entire pregnancy*	Number of days with rainfall >5mm	2.53**	0.87–4.19	6.59**	1.50–11.68
	Mean temperature (°C)	45.32	-17.51–108.15	**154.42†**	-23.97–332.82

***p<0.001

**p<0.01

*p<0.05

†p<0.10

N.B. Models include all control variables from the baseline model (Table 4). Coefficients and confidence intervals for control variables are not shown, but did not substantively differ from the baseline model. Each model includes both rainfall and temperature variables together in the model. Models are stratified by trimesters and population subsets

We also tested for interaction between sex and meteorological values and found no significant interactions. Lastly, we included dummy variables for month in the full models and observed no changes in the patterns of association.

## 4. Discussion

To our knowledge, this is one of the first studies to examine the effects of specific perinatal meteorological exposures on birth weight in a vulnerable sub-Saharan Africa population, and the first to consider effect modification by existing social gradients in health in a specific regional setting. We find comparable results to Grace, Davenport [17] with respect to the direction of associations between precipitation predictors and birth weight, but note that the magnitude of association is greater in our study, indicating that precipitation may have a greater effect on birth weight in this rural population compared to the continental average. Our results are also consistent with our hypothesis that meteorological impacts incurred in the third trimester have the most significant impact on birth weight. The positive effect of precipitation on birth weight might be related to, at least partially, harvest cycles and maternal nutritional status, as food may be more plentiful in the rainy season.

Season of birth did not emerge in the initial models as a significant determinant of birth weight. However, when we stratified the sample by season of birth, the associations between meteorological exposures and birth weight were inconsistent across babies born at different times of year. Indeed, only those born between June and August showed significant associations between in utero temperature and precipitation exposures; those born between September and November showed a significant exposure only between third trimester average temperature exposure and birth weight. This heightened sensitivity to meteorological exposures for July through August births (the driest and hottest season) could be linked to harvest cycles in the region—women who give birth in this season may benefit more from increased temperatures and increased rainfall in their third trimester as these conditions could increase the harvest yield in a time of year when food is most scarce. The third trimester is a crucial period for increasing birth weight, leading us to conjecture that the reverse trends observed for second trimester exposures may be residual effects related to a preferencing of the most optimal conditions during the last trimester.

The explanation of the positive effect of temperature on birth weight in season 4 (September to November) is less obvious, and may be related to the effects of temperature on local harvest cycles (perhaps due to a latent effect of sensitivity to meteorological exposures during the preceding season) or potentially to more direct physiological effects of ambient temperature on foetal development [14, 58]. Variation in temperature in the region is relatively minimal; during the study period average daily temperature ranged between 16°C to 23°C. While we identify significant associations, these do not imply causation or distinguish the potential causal mechanisms by which meteorological variables might affect birth weight. In this context, weather may be affecting birth weight directly through nutritional resources, or indirectly through impacts on gestational age. We cannot infer, for example, that warming temperatures projected for the region [43, 44] will lead to increased birth weights. Understanding the causal pathways through that may be contributing to the association between temperature and birth weight would benefit from further exploration, particularly from a qualitative perspective.

In the non-Indigenous subset, it was primarily weather exposures incurred in the third trimester that were associated with birth weight. However, the effect of mean temperature exposure appeared to be important throughout pregnancy in several models examining only Indigenous Batwa cases. We found some variation in the associations between meteorological exposures and birth weight among Batwa compared to the non-Indigenous population. This may reflect modification of meteorological impacts on birth outcomes by ethnicity among mothers living in the same region and with access to similar health services.

The differential impacts of temperature on birth weight between the Indigenous and non-Indigenous subsets may reflect differences in agricultural cycles between the two populations. Patterson et al. (Under review) found, for example, that the Batwa seasons of harvest and famine begin earlier than neighbouring non-Indigenous populations in Kanungu District due to high impoverishment and limited historic experience with agriculture. Thus, the direct physiological effects of ambient temperature on birth weight may supersede those of variation in food availability according to seasonal precipitation in this population. This result is consistent with findings from Grace, Davenport [17] that showed the effects of temperature being significant in non-agriculturalist livelihood groups only, as the Batwa are still adapting to the agrarian lifestyle that has been imposed upon them [28]. Maternal height and weight are important determinants of birth weight [15], and Batwa are an Indigenous pygmy tribe characterized by small stature. While this could explain significant differences in birth weight by ethnicity (mean birth weight was 3090.0g for non-Indigenous infants and 2797.0g for Batwa infants, t = 3.80 (32.8), p<0.01), it would not explain the differences in the nature of the meteorology-birth weight relationship between the two groups.

One of the chief limitations to this study is its reliance on hospital births, a sample which is likely not representative of all births in the area and thus limits the generalizability of our findings. In 2013–2014, for example, only 1264 (42%) of the estimated 2958 deliveries in the catchment area occurred in the hospital [40]. This split between mothers giving birth at home versus in the hospital is a potential source of endogeneity due to unobserved variables affecting a mother’s choice to give birth in hospital. We do not know if healthier, wealthier mothers are self-selecting to deliver at the hospital, or if higher risk pregnancies are referred to a hospital for birth by community health workers and smaller clinics; both trends may be present in the dataset, though it is more likely that the former predominates over the latter. Given the impoverishment of the region and cost associated with hospital services, we expect that our sample is more likely a subset of wealthier/higher SES mothers, and that the results presented here are likely underestimates compared to the general community population. Findings from this study cannot, however, be appropriately extrapolated from mothers choosing hospital-based births to the general population. Strand, Barnett [59] critique the use of retrospective cohorts for assessing seasonal patterns in birth outcomes due to the potential for a “fixed cohort bias”—the exclusion of shorter pregnancies at the beginning of studies and longer pregnancies at the end—though they allow for the necessity of such studies in some contexts; in this instance our sample size would have been severely compromised by their recommended cut-offs. Several control variables absent from the database would have enhanced this analysis, including maternal height and weight, more complete records of malarial infection throughout during pregnancy, maternal nutritional status, SES, and consistent estimates of gestational age via ultrasound dating early in pregnancy. Measures used for ascertaining gestational age in our study included not only ultrasound-based dating but also LMP- and fundal height-based dating. Gestational age based on LMP has been criticised for recall bias [60]. Fundal height can only offer a crude approximation of actual gestational age compared to ultrasound dating [61, 62], especially when gestational age is derived from only one point measure of fundal height [63]. In addition to the error inherent to these measures individually, combining gestational age data from a range of measures (including fundal height, LMP recall, and ultrasound dating) is problematic for comparison to any birth weight for gestational age distribution. We attempt to mitigate these limitations with sensitivity analyses for the subset of births for which GA was determined by ultrasound, which showed that model behaviour for this subset was generally consistent with trends observed for the full dataset. Including a GA variable in the models does pose a risk of over-controlling given the overlap in the causal pathways of GA and birth weight, but our sensitivity analyses of term births and models without GA controls were robust. In this case, we elected to look specifically at the non-GA related associations with birth weight and thus controlled for it. The associations between GA and meteorological exposures, as well as the causal pathways contributing to these associations, merit further consideration in future research. The sample size of the Batwa mothers is a further limitation to our study, however it is reflective of the demographic composition of the region, of which Indigenous persons make up 1% of the population. The statistical power of this sample is low, which translates to an unavoidable lack of precision in the Batwa results.

Our results highlight heterogeneity in the vulnerability of infant health to meteorological variation in different contexts, with implications for climate change impacts and adaptation research. The health effects of climate change will manifest along existing social gradients, with gendered impacts on nutrition highlighted as an area of particular concern since women and girls’ nutrition is often deprioritized relative to their male relatives [64]. Our results indicate that while there is a significant effect of meteorological variation on birth weight; these effects may vary along social gradients. This implies that interventions to reduce existing social gradients may be of sufficient magnitude to offset some health impacts predicted under climate change.

Pregnancy results in increased energy consumption and increased nutritional needs [65], rendering pregnant women especially vulnerable to periods of food scarcity anticipated with the effects of climate change [7]. This study highlights the importance of the third trimester as most vulnerable to meteorological effects on birth weight, consistent with other research [66–68]. Given that this is likely linked to maternal nutritional needs, this information identifies women in their third trimesters as an important group in need of additional nutritional resources, particularly in periods of little rain. While there does not appear to be a most optimal season in which to give birth, there may be increased sensitivity to meteorological exposures in the June to November period. Monitoring weather conditions for women expected to deliver in this window could be important for targeting nutritional interventions. Indigenous Batwa mothers may be at magnified risk—beyond existing infant health disparities—during periods of cold, particularly in the third trimester. The health inequities the Batwa face more broadly put the most vulnerable among them—pregnant women and infants—at greater risk, and interventions, both immediate and upstream, are needed to eliminate these inequities.

The results of this study are of particular relevance to maternal health services at Bwindi Community Hospital, which aims to decrease maternal and child mortality rates by 25% by 2019 [40]. As climate in the Kanungu region becomes less predictable with more extreme rainfall [43, 44] and drought [46] events projected, agricultural production in the Kanungu region could be threatened [34]. Planning for these changes and developing interventions responsive to both the current and future needs of pregnant women and newborns in the region is a health priority and grand challenge.
